# In Vitro Experimental and Numerical Simulation Study on the Influence of Uniaxial Cyclic Compression on Cytoskeletal Structure

**DOI:** 10.3390/bioengineering12121317

**Published:** 2025-12-02

**Authors:** Lu Yu, Jingyi Jia, Tianyi Zhang, Yifei Yao

**Affiliations:** 1 School of Biomedical Engineering, Shanghai Jiao Tong University, Shanghai 200030, China; 641032847@sjtu.edu.cn (L.Y.); jiajingyi@sjtu.edu.cn (J.J.); 2 Engineering Research Center of Digital Medicine and Clinical Translation, Ministry of Education, Shanghai Jiao Tong University, Shanghai 200030, China; 3 Clinical College, Anhui Medical University, Hefei 230032, China; zty070330@163.com

**Keywords:** cell biomechanics, actin filament reorientation, cyclic compression, finite element analysis

## Abstract

While research on cellular responses to cyclic compression has predominantly focused on proliferation and differentiation, changes in cell orientation and force distribution within the cytoskeleton represent crucial biomechanical aspects that remain less explored. This study aimed to design a programmable device for applying uniaxial cyclic compression to cells and analyze actin filament reorientation following specific compression regimens. A programmable device was developed to apply uniaxial cyclic compression. A finite element model of a viscoelastic cell incorporating actin filaments was developed to evaluate cell membrane strain. Statistical analysis included Pearson correlation to assess the relationship between actin filament orientation and membrane strain, following normality confirmation with the Kolmogorov–Smirnov test. Student’s *t*-test and one-way ANOVA were used to assess significance between groups. A strong positive correlation was found between the average/peak maximum principal strain on the cell membrane and the angle of actin filaments relative to the cell long axis (r = 0.96, *p* < 0.05; r = 0.94, *p* < 0.05, respectively). Cyclic compression reduced the maximum principal strain by reversing the actin filament orientation observed under static compression. This correlated with a significant decrease in cell mortality. Cyclic compression reduces the maximum principal strain on the cell membrane via reorientation of actin filaments, suggesting a cytoprotective effect. These findings provide insight into biomechanical adaptive mechanisms of cells under cyclic compression and could inform the design of bioreactors and rehabilitation devices.

## 1. Introduction

Mechanical stimuli are important regulators for cells to proliferate [1], differentiate [2], and migrate [3]. Such mechanical regulation occurs because cells can sense and adapt their internal prestress to respond to environmental mechanical signals such as compression, tension, and shear [4]. An important manifestation of this adaptation involves cellular morphological and structural changes, among which the reorientation of the cell cytoskeleton in response to mechanical stimuli is particularly significant [5]. The eukaryotic cell cytoskeleton, comprising interconnected networks of microtubules, actin filaments, and intermediate filaments [6], provides the structural integrity necessary to resist deformation. Recent studies have demonstrated that both internal and external physical forces modulate local mechanical properties and cellular behavior via the cytoskeleton [7,8].

Cyclic compressive stimulation was found to be useful in cell proliferation, differentiation [9], and determination of cell fate. Yao et al. [10] discovered that cyclic compression enhanced the ability of muscle cells to resist mechanical stimuli. Huang et al. [11] found that cyclic compressive loading could promote the chondrogenesis of rabbit bone marrow mesenchymal stem cells (BM-MSCs) by inducing the synthesis of TGF-β1, which can stimulate the BM-MSCs to differentiate into chondrocytes. However, limited studies have focused on the effect of cyclic compressive stimulation on cellular subcomponents, particularly in muscle cells.

Complementing biological investigations, researchers have employed finite element (FE) models to study force distribution within the cytoskeleton and the mechanisms underlying cell reorientation. FE modeling is an effective tool for simulating cellular biomechanics, significantly reducing the uncertainty inherent in experimental measurements. While some models represent cells as homogeneous viscoelastic materials [12,13], others utilize tensegrity structures to model cytoskeletal subcomponents [14,15]. In our previous FE study of cellular response to cyclic compression, we demonstrated that cyclic compression could decrease the average tensile strain on the cell membrane, potentially mitigating cell damage [16]. Nevertheless, cytoskeletal mechanics play a fundamental role in numerous cellular processes.

While prior studies focused on proliferation/differentiation under cyclic compression [17,18], the biomechanical adaptations in cytoskeletal orientation remain underexplored. To bridge this gap, we designed a programmable device for applying uniaxial cyclic compressive stress to cells and quantify the reorganization of the actin cytoskeleton following compression exposure. Furthermore, by developing a finite element model of a viscoelastic cell incorporating the cytoskeleton, we analyzed cell membrane strain under cyclic compression. Integrating in vitro experimental results with FE simulations provides deeper insights into the mechanisms of cellular adaptation to cyclic compression.

## 2. Materials and Methods

### 2.1. Design of the Cyclic Compression Device

**Figure 1:**
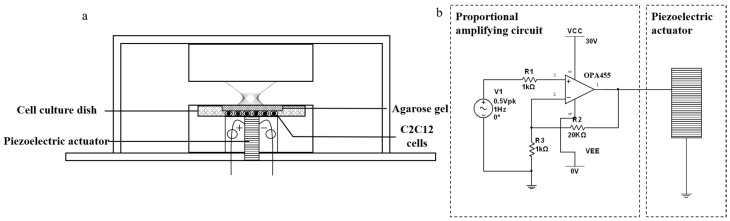
Conceptual design diagram of the device: (**a**) physical framework; (**b**) piezoelectric ceramic driving circuit.

The cyclic compression device was composed of a piezoelectric actuator (with a drive circuit), a compression application module, and a cell culture dish placement tank. The design of the device is illustrated in Figure 1a,b. The compression application module was positioned at the top of the cell culture dish placement tank, below which was located the piezoelectric actuator (Figure 1a). The drive circuit was connected to the piezoelectric actuator (Figure 1b).

The working principle of the device was that the compression application module applied static compression (prestress) to cells through a layer of 0.5% agarose gel placed above the cells to ensure that all the cells below the gel were under compression. Agarose gels used as the medium to transmit cyclic compressive stimulation to the cells were prepared by dissolving 100 mg or 200 mg of agarose in 20 mL of phosphate-buffered saline (PBS). The suspension was heated in a microwave oven for 2 min until boiling and until no white suspended particles were visible. The hot solution was then poured into a 100 mm culture dish and allowed to solidify at room temperature. Cylindrical agarose specimens with a diameter of 14.55 mm and a thickness of 3 mm were cut from the gel and stored in PBS prior to testing. The mechanical properties of the agarose gels were characterized using a material testing machine (HZ-1007E, Dongguan Lixian Instrument Co., Ltd., Dongguan, China) in unconfined compression. A constant displacement rate of 1 mm/min was applied, and the displacement accuracy of the apparatus is specified by the manufacturer to be within ±0.2% of the indicated value. Engineering stress–strain curves were derived from the force–displacement data, and Young’s modulus was calculated from the linear portion curve fitting of the stress–strain response. The drive circuit of the piezoelectric actuator generated sinusoidal stimulation to the piezoelectric actuator, which further applied amplified sinusoidal vibration to the bottom of the adherent cells. The combined action of the static prestress (applied via agarose gel) and the dynamic vibration (delivered by the piezo actuator) resulted in a net cyclic compressive load on the cells, with the gel ensuring uniform stress distribution.

### 2.2. Myoblast Culture

Undifferentiated C2C12 mouse skeletal myoblasts (ATCC, 10801 University Blvd. Manassas, VA, USA) were cultured in a growth medium comprising Dulbecco’s modified Eagle medium (DMEM, with 4.5 g/L glucose, Gibco, Life Technologies, Grand Island, NY, USA), 10% fetal bovine serum (FBS, Gibco, Life Technologies, Grand Island, NY, USA), and 1% penicillin–streptomycin (Gibco, Life Technologies, Grand Island, NY, USA). Cell passaging was performed every 2–3 days by trypsinizing the myoblasts with 0.25% trypsin-EDTA (1×, Gibco, Life Technologies, Grand Island, NY, USA). The cells were then incubated in a humidified incubator at 37 °C and 5% CO_2_.

### 2.3. Cell Compressing Experiments

C2C12 myoblasts (passages 15–16) were cultured at low density (∼2 × 10^3^ cells/cm^2^) for high-resolution actin filament imaging and normal density (∼10^5^ cells/cm^2^) for viability assessment via Calcein-AM/PI staining. Static compression was applied to the cells with different periods (0, 10 min, 20 min, 30 min, and 60 min) and amplitudes (75 Pa, 100 Pa, 125 Pa, 150 Pa, and 175 Pa). Cyclic compression was applied to the cells with sinusoidal compressive stress of 100 ± 50 Pa at 0 Hz (static compression of 100 Pa), 0.25 Hz, 0.5 Hz, 1 Hz, and 10 Hz for different periods (0 min, 20 min, and 60 min).

### 2.4. Fluorescence Staining and Imaging

Actin filaments of C2C12 myoblasts were stained with rhodamine-phalloidin (excitation/emission: 493/517 nm, molecular probesTM, Abacam, 152 Grove Street, Suite 1100, Waltham, MA, USA) to obtain actin filaments and the cell outline at room temperature for 1 h. Stained cells were then observed using a confocal microscope (Leica Camera AG, SP 9, Am Leitz-Park 6, Wetzlar, Germany) with a 40× immersion oil objective lens. Sequential confocal z-stack images were acquired at 600 nm intervals. C2C12 cell viability tests under static and cyclic compression were carried out using Calcein-AM/PI staining (Calcein-AM excitation/emission = 494/517 nm, PI excitation/emission = 535/617 nm, Beyotime, 1500 Xinfei Road, Shanghai, China) at 37 °C in the dark for 30 min. The images of stained cells were captured with a fluorescence microscope (Leica Camera AG, SP 9, Am Leitz-Park 6, Wetzlar, Germany).

### 2.5. Quantification of Cell Reorientation and Statistical Analysis

To quantify the cell reorientation in response to static and cyclic compression, the main calculation involved determining the average angle between each actin filament and the long axis of the cell to quantify changes in actin filament orientation in response to varying cyclic compression.

**Figure 2:**
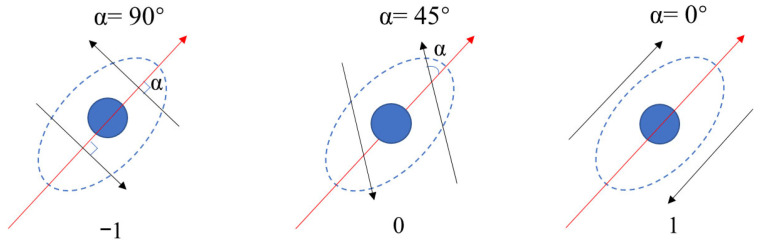
Definition of the mean orientation value calculated by the angle between actin filaments and the cell long axis.

The core idea of the quantification algorithm is depicted in Figure 2, where the red arrow represents the long axis of the cell, the black arrows represent the cell’s actin filament, and α represents the angle between them. When the long axis and the actin filament were parallel (α = 0°), the quantified value for actin filament orientation was defined to be 1. When they were perpendicular (α = 90°), the quantified value was −1. When the angle was between 0° and 90°, the quantified value for actin filament orientation ranged between 1 and −1, shown in Formula (1).

mean orientation value = 1 − α/45°(1)

**Figure 3:**
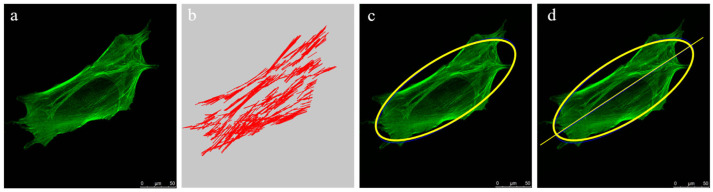
Implementation steps of quantification algorithm for cell actin filament orientation: (**a**) actin filaments staining; (**b**) actin filaments detection; (**c**) ellipse fitting; (**d**) long axis calculation.

The implementation steps for the actin filament quantification algorithm are shown in Figure 3. The outlines of the stained cells were recognized and fitted into ellipse shapes first via the “findContours” function in OpenCV to determine the cell’s long axis. The detection of actin filaments was further achieved via “canny” and “HoughLinesP” functions in OpenCV.

### 2.6. Three-Dimensional Finite Element Model of Myoblast

A cell FE model containing the cytoplasm, the nucleus, the actin filament, and the membrane was developed to simulate cell membrane tensile strain under static and cyclic compression. The model construction and validation process were shown in our previous work [16].

The mechanical properties of both the cytoplasm and nucleus were considered isotropic, homogeneous, and viscoelastic, listed in Table 1 [19,20]. Finite element simulations were performed using Abaqus 2021 (Dassault Systèmes, Vélizy-Villacoublay, France). The cytoplasm and nucleus were meshed with 10-node quadratic tetrahedral solid elements (Abaqus element type C3D10). A tie constraint was assigned between the outer surface of the nucleus and the inner face of the cytoplasm complex. The cell membrane was represented by a single layer of elements at the apical surface of the cell. The nodes at the base of the myoblast model were fixed in all six degrees of freedom. The optimal number of elements was determined to be 14,551 for the nucleus and 7393 for the cytoplasm complex respectively after mesh convergence tests.

The boundary conditions involved applying a sinusoidal compressive stress of 100 ± 50 Pa at 0 Hz (equivalent to a static compression of 100 Pa) as well as at frequencies of 0.25 Hz, 0.5 Hz, 1 Hz, and 10 Hz. The cyclic stimulus was uniformly applied to the nodes on the cell membrane of the FE model.

**Figure 4:**
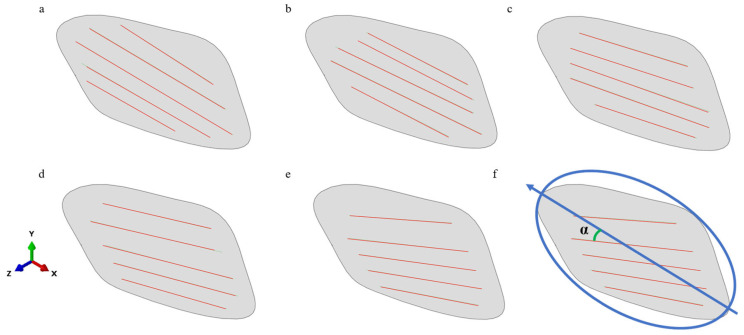
Cell actin filament model under static compression of 100 Pa and different times: (**a**) control; (**b**) static 100Pa, 10 min; (**c**) static 100 Pa, 20 min; (**d**) static 100 Pa, 30 min; (**e**) static 100 Pa, 60 min; (**f**) filament directionality adjustment based on α.

The orientation of actin filaments in the FE model was defined based on the experimentally measured mean orientation values corresponding to each compression condition. Specifically, lines representing actin filaments were incorporated at the basal surface of the cell model, with their directionality adjusted to match the quantified orientation angle (α) derived from experiments, shown in Figure 4.

### 2.7. The Relationship Between Maximum Principal Strain on the Cell Membrane and Cell Death Rate

To further explore the relationship between the maximum principal strain on the cell membrane and cell death under cyclic compression, it was assumed that cells would be damaged beyond a tensile strain threshold of cell membrane elements (20%) [21]. The percentage difference between damaged elements on the cell membrane and the cell death rate were defined as follows:

Percentage difference of DE = (1 − DE_cyclic/_DE_static_) × 100% (2)

Cell death rate = ND/T × 100%(3)

where DE stands for damaged elements, ND the number of dead cells, and T the total number of cells.

### 2.8. Data Analysis

The results were presented as means ± standard deviation (SD) of at least three independent samples. Statistical significance between groups was assessed using Student’s *t*-test and one-way analysis of variance (ANOVA). Pearson correlation analysis was employed to assess the relationship between the actin filament orientation angle and the average/peak maximum principal strain on the cell membrane, following confirmation of data normality using the Kolmogorov–Smirnov test. * *p* < 0.05 indicated a statistically significant difference, while ** *p* < 0.01 and *** *p* < 0.001 indicated highly significant differences.

## 3. Results

### 3.1. Characterization of 0.5% Agarose Gels

**Figure 5:**
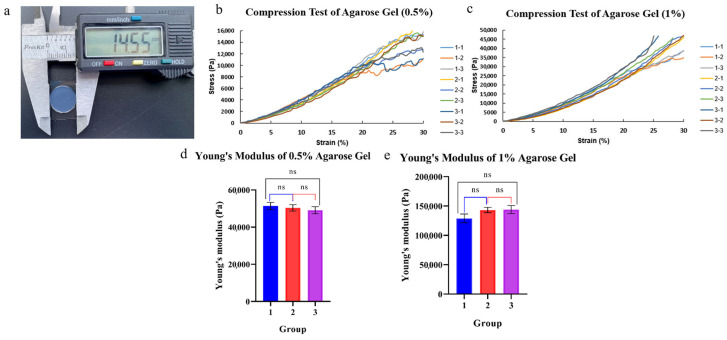
Preparation and compression test of agarose gel: (**a**) agarose gel used in compression test; (**b**) compression test results of 0.5% agarose gel; (**c**) compression test results of 1% agarose gel; (**d**) statistical analysis of compression test of 0.5% agarose gel; (**e**) statistical analysis of compression test of 1% agarose gel; ns: *p* > 0.05.

Three groups of 0.5% and 1% cylindrical agarose gels were prepared and their Young’s moduli were tested, as shown in Figure 5a,b. There was no significant difference between the three groups, and Young’s modulus of 0.5% and 1% agarose gel was 50.3 ± 1.8 kPa and 138.7 ± 8.7 kPa respectively. The 0.5% concentration was selected as it facilitated effective transmission of compressive stress while minimizing cellular adhesion.

### 3.2. Validation of the Cyclic Compression Device

**Figure 6:**
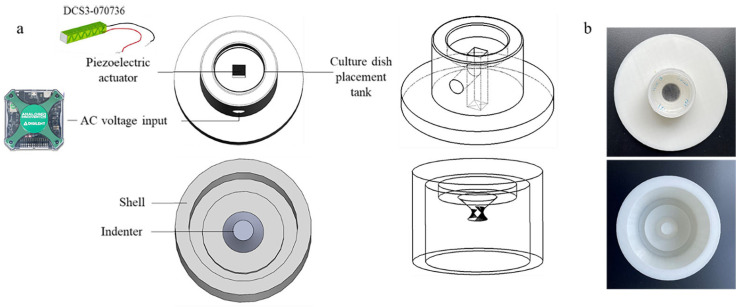
Device framework design and entity diagram: (**a**) device diagram based on SolidWorks 2021 design; (**b**) device entity diagram using PLA material 3D printing.

**Figure 7:**
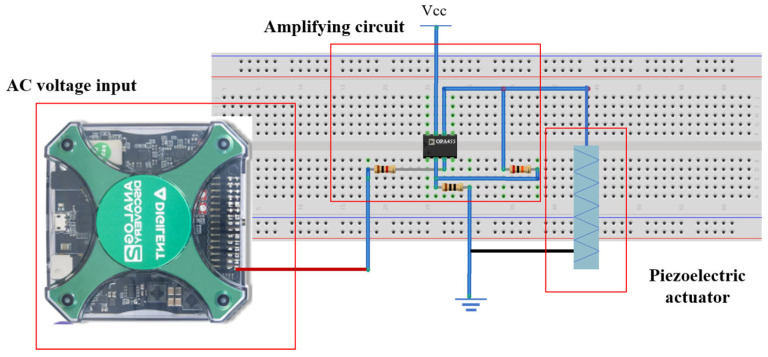
Physical diagram of the device circuit.

The fabrication results are shown in Figure 6a,b. The device was designed in SolidWorks 2021 (Dassault Systèmes, SolidWorks Corporation, Waltham, MA, USA) and fabricated via 3D printing (Raise 3D Pro 2, Tesla, Irvine, CA, USA) using 1.75 mm PLA (polylactic acid) filament. A drive circuit for the piezoelectric actuator was constructed, achieving an amplification factor of 20 (Figure 7).

The validation of the designed cyclic compression device was conducted to obtain a clear correspondence between the input and output of the device. The input parameters of the device included the AC voltage amplitude (V_amplitude_), AC voltage offset (V_offset_), and frequency (f), all of which would affect the range of cyclic stimulation applied to cells by the device. Therefore, the relationship needed to be fitted as follows:

V_output_ = V_amplitude_ sin ω (t + t_0_) + V_offset_ (4)

ω = 2πf(5)

**Figure 8:**
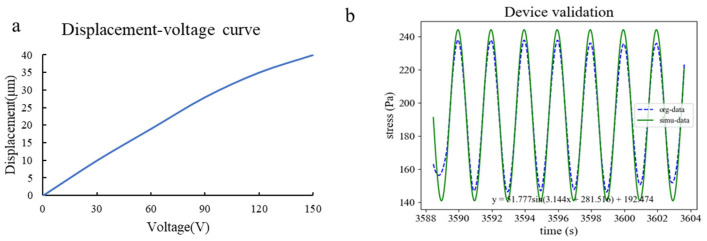
Feasibility verification of the device: (**a**) characteristic curve of the piezoelectric ceramic driver; (**b**) sine wave fitting based on test results.

Firstly, the characteristics of the piezoelectric actuator used in the device are shown in Figure 8a, with a displacement of 10 μm under 30 V voltage. The strain of the 0.5% agarose gel could be determined when the upper surface was fixed and the lower surface stimulated by the actuator. The compressive stress exerted on cells by the gel could be calculated using Young’s modulus obtained from the compression test (Section 3.1). By adjusting the V_amplitude_ and V_offset_ of the piezoelectric actuator’s vibration, the amplitude and offset of the cyclic compression can be controlled to achieve cyclic compression on the cells.

A real-time stress–duration curve of the piezoelectric actuator’s cyclic vibration was obtained by the material testing machine (Dongguan Lixian Instrument Technology Co., Dongguan, China). The fitting result yielded a sinusoidal curve described by

y = 51.777 sin (3.144 x + 281.516) + 192.474(6)

where y stands for stress (Pa) and x stands for time (s), which confirms the device’s ability to apply cyclic compression stress to cells, shown in Figure 8b.

### 3.3. Cell Viability Tests Under Static and Cyclic Compression

**Figure 9:**
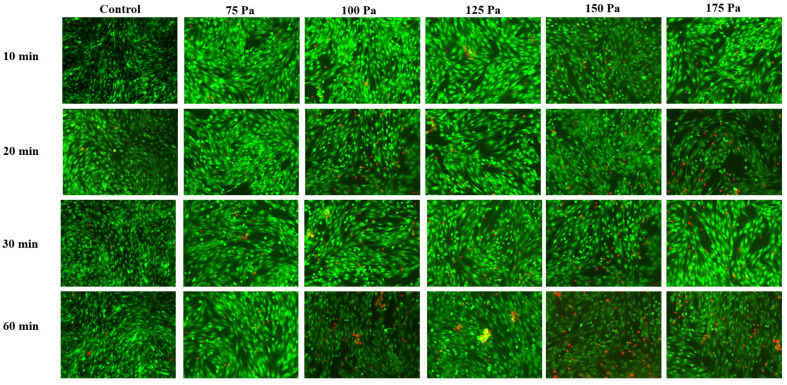
Calcein-AM/PI cell viability staining results under different time and static compression amplitudes.

**Figure 10:**
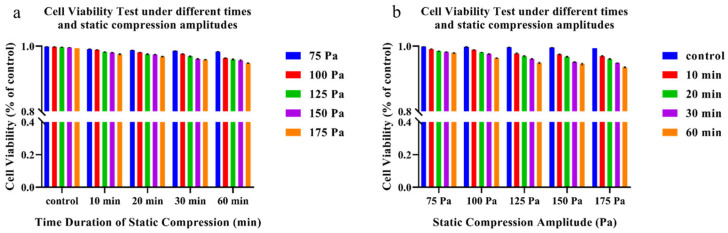
Statistical analysis of Calcein-AM/PI cell viability under different time and static compression amplitudes. (**a**) The time-dependent result; (**b**) the static compression amplitude-dependent result.

The results of the cell viability test under static compression (Figure 9 and Figure 10) showed that with a constant compression duration, as the static compression magnitude increased, the cell death rate significantly increased. Similarly, with a constant compression intensity, as the compression duration extended, the cell death rate increased. Static compression of 100 Pa was chosen to further compare the differences between static compression and cyclic compression.

**Figure 11:**
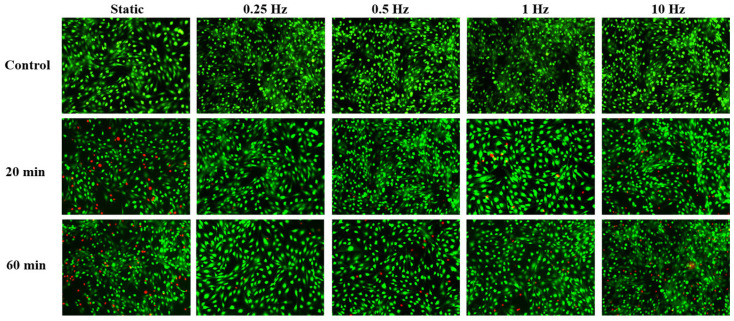
Calcein-AM/PI cell viability staining results under different time and cyclic compression frequencies.

**Figure 12:**
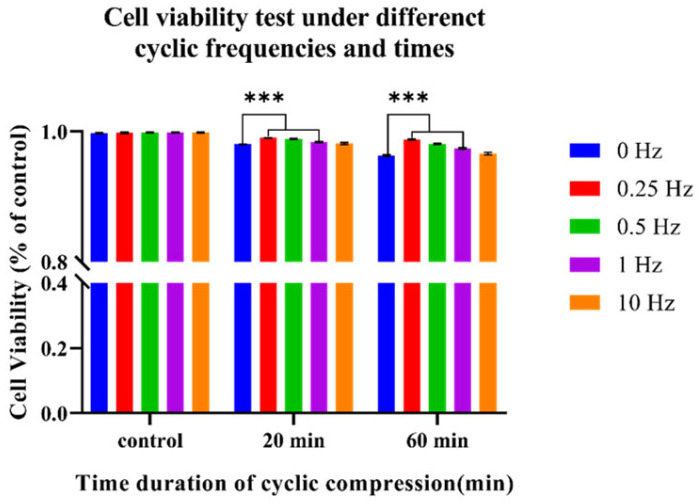
Statistical analysis of Calcein-AM/PI cell viability under different times and frequencies of cyclic compression. n = 3, *** *p* < 0.001.

The results of the cell viability test under cyclic compression (amplitude of 100 ± 50 Pa, frequency of 0.25 Hz, 0.5 Hz, 1 Hz, and 10 Hz) showed that the cell death rate decreased compared with static compression, especially under 0.25 Hz, shown in Figure 11 and Figure 12.

### 3.4. Quantification of Actin Filament Orientation

**Figure 13:**
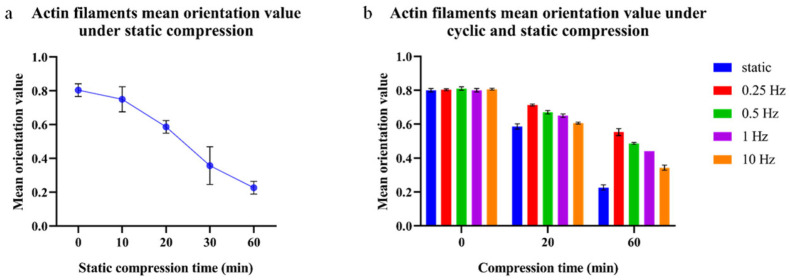
Comparison of cell actin filaments’ mean orientation values under cyclic compression amplitude of 100 Pa ± 50 Pa, time of 20 to 60 min, frequency of 0.25, 0.5, 1, and 10 Hz, and static compression at 100 Pa.

The calculated results of cell actin filament orientation under static and cyclic compressions are presented in Figure 13. Actin filament orientation was consistent across cells in the control group (no compression). Furthermore, the same staining and calculation were performed for cells subjected to 100 Pa static compression for 10, 20, 30, and 60 min. The results showed that as the compression duration increased, the mean orientation value decreased, indicating that the angle between cell actin filaments and the cell long axis gradually increased. As for the cyclic compression group, the mean orientation value increased compared with the static group after the same compression time, indicating that the angle between actin filaments and the cell long axis decreased. The most significant reduction was at 0.25 Hz. Representative fluorescence images and images with detected actin filaments for the different time points and frequencies are provided in Supplementary Figures S1–S5.

### 3.5. Maximum Principal Strain on the Cell Membrane Through FEA

**Figure 14:**
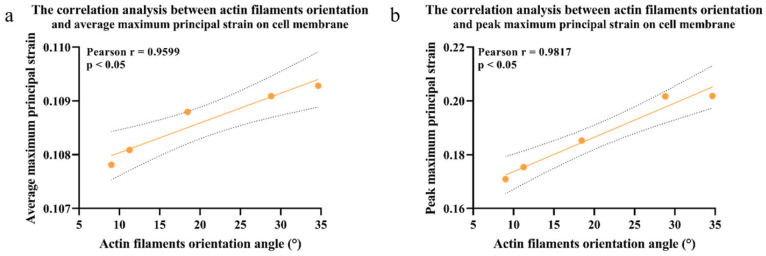
Correlation analysis of actin filaments’ orientation angle and the (**a**) average tensile strain on the cell membrane surface; (**b**) maximum tensile strain under static compression of 100 Pa for different times. The 95% confidence interval is represented by dashed lines in the graph.

The correlation analysis between the average and peak maximum principal strains on the cell membrane and the change in cell actin filament orientation is shown in Figure 14. The Pearson correlation analysis results indicated a positive correlation between the angle of cell actin filaments and the average principal strain on the cell membrane surface (r = 0.96, *p* < 0.05). There was also a positive correlation between the angle of cell actin filaments and the maximum principal strain on the cell membrane surface (r = 0.94, *p* < 0.05). As the static compression duration increased, the angle between cell actin filaments and the cell long axis also increased, and the average/maximum principal strains on the cell membrane surface increased, indicating that the cells were adversely affected by static compression.

**Figure 15:**
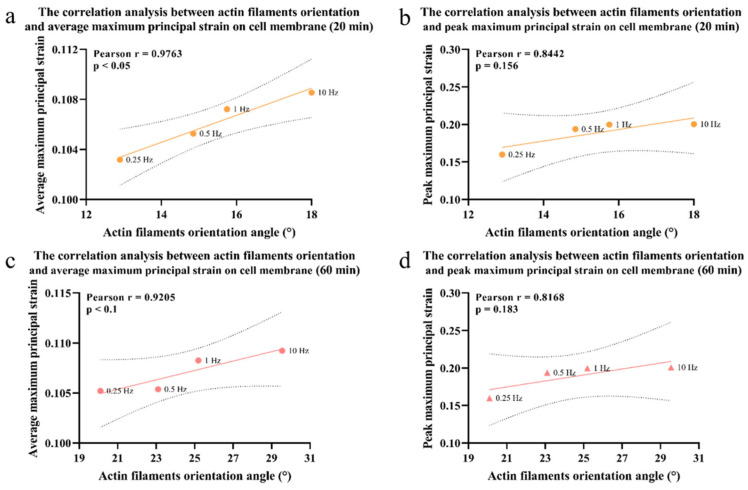
Correlation analysis of actin filaments’ orientation angle and the (**a**) average tensile strain on cell membrane under cyclic compression for 20 min; (**b**) maximum tensile strain on cell membrane under cyclic compression for 20 min; (**c**) average tensile strain on cell membrane under cyclic compression for 60 min; (**d**) maximum tensile strain on cell membrane under cyclic compression for 60 min. The 95% confidence interval is represented by dashed lines in the graph.

**Figure 16:**
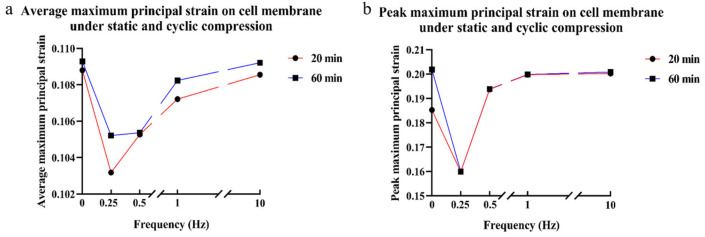
Cell membrane tensile strain under cyclic compression of 100 Pa ± 50 Pa at different frequencies and times; (**a**) average tensile strain value; (**b**) maximum tensile strain value. The red line with circular symbols represents 20 min, whereas the blue line with square symbols represents 60 min.

The actin filaments under cyclic compression showed the reversed results. As the cyclic compression duration increased, the angle between cell actin filaments and the cell long axis decreased, and the average/maximum principal strains on the cell membrane surface decreased, shown in Figure 15 and Figure 16. Representative contour plots of the maximum principal strain distribution and its peak value on cell membrane under 20/60 min static and cyclic compression are shown in Supplementary Figures S6 and S7.

### 3.6. The Relationship Between Maximum Principal Strain on the Cell Membrane and Cell Death Rate

**Figure 17:**
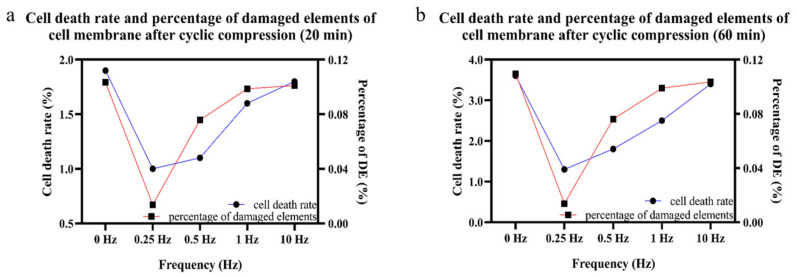
Comparison between the percentage of damaged elements of the cell membrane (damage threshold of 20%) and cell death rate under static and cyclic compression at different times and frequencies, (**a**) compression for 20 min; (**b**) compression for 60 min. The blue line with circular symbols represents cell death rate, whereas the red line with square symbols represents percentage of damaged elements.

The result showed that the change in the percentage of damaged elements on the cell membrane was consistent with the trend of the cell death rate, both reaching their minimum values under cyclic compression at 0.25 Hz, shown in Figure 17.

## 4. Discussion

The pervasive influence of mechanical stimuli on cellular behavior—spanning proliferation, differentiation, and cytoskeletal reorganization—represents a fundamental axis of mechanobiology. While prior studies predominantly focused on biochemical pathways [1,2,3,4,22], this work elucidated how uniaxial cyclic compression induces actin filament realignment to mitigate mechanical damage—a biomechanical adaptation distinct from canonical signaling cascades. Our findings aligned with emerging evidence that cytoskeletal reorientation serves as a primary adaptive strategy against sustained mechanical stress [5,23]. The observed actin realignment (filament orientation angle decreases) under cyclic loading parallels Huang et al.’s report of cytoskeletal preconditioning in chondrocytes [11].

In this study, 0.5% agarose was selected as the loading interface based on its use as a biologically inert, low-adhesion overlay in cell mechanobiology studies for applying mechanical stimuli. This approach leverages the inherent bio-inertness of agarose, which lacks specific biochemical motifs to promote strong cell adhesion [24,25]. Our mechanical characterization further informed this choice, as lower agarose concentrations proved too fragile for consistent mechanical loading, while 1.0% agarose, despite its mechanical robustness, exhibited undesirable macroscopic stickiness during handling. While we did not directly quantify hydrogel–cell adhesion in this study, the selection of 0.5% agarose is consistent with the goal of creating a compliant interface that transmits mechanical stress without inducing strong, confounding hydrogel–cell adhesive interactions.

The programmable compression device developed herein addresses longstanding limitations in delivering physiologically relevant cyclic compressive stresses or strains. Unlike commercial systems constrained by fixed loading profiles [26] our agarose-based stress-transducing layer enables precise transmission of low-magnitude stresses (75–175 Pa) while reducing the influence of hydrogel–cell adhesion artifacts—a design advance corroborated by Singh and Chanda’s framework for biomimetic loading interfaces [27]. This is an expected advantage based on the device design, in which cells are cultured on the bottom of the culture dish with a compliant agarose hydrogel layer placed on top to transmit compressive loads, and on prior knowledge that such hydrogel-based interfaces can better mimic soft tissue environments and reduce hydrogel–cell adhesion artifacts. Qualitatively, we observed in bright field imaging that cells maintained confluency throughout the experiments, suggesting minimal gross detachment or interfacial disruption. Integrating this technology with computational modeling reveals a sequential biomechanical response, where cyclic compression promotes actin realignment, leading to reduced membrane strain and consequently enhanced viability. The inverse correlation between filament orientation angle (α) and maximum principal strain substantiates Yao et al.’s findings that cyclic compressive stimulation strengthens cells [10].

Our FE model, incorporating actin filament directionality derived from experimental α values, provides unprecedented resolution of subcellular strain distributions. The positive α-strain correlation (r = 0.96, *p* < 0.05) validates tensegrity principles in which filament orientation dictates force redistribution [14,15,28]. Specifically, perpendicular actin alignment under static compression concentrates strain at membrane anchor points, which was consistent with Bansod et al.’s prediction of focal adhesion overload in misoriented cytoskeletons [29]. Conversely, cyclic loading promotes parallel filament arrangements that dissipate stresses longitudinally, reducing peak membrane strains below the 20% damage threshold established for myoblast membranes [21]. This mechanism may explain a cell-type-dependent response in the literature, where Kontogianni et al. [18] observed reduced osteogenesis at high compression frequencies, whereas we demonstrate maximal cytoprotection at 0.25 Hz—a divergence attributable to cell-type-specific cytoskeletal architectures and differential strain tolerances.

The paradigm of “cytoskeletal realignment as mechanoprotection” extends beyond myoblasts to inform tissue engineering. In mechanosensitive tissues like cartilage, where cyclic compression enhances chondrogenesis [11], future studies could investigate whether actin reorganization synergizes with TGF-β upregulation in mesenchymal stem cells. Similarly, cancer metastasis studies linking cell softening to invasion [12,30] might integrate compression-induced actin bundling observed here. Methodologically, our combined experimental–computational approach advances beyond pure in silico studies [14,31] by empirically constraining FE parameters, a strategy endorsed by Blache et al. for studying mechanotransduction [8].

These findings crystallize three translational principles. First, 0.25 Hz could provide optimum bioreactor designs for muscle tissue engineering. Second, membrane strain thresholds (≤20%) may provide quantifiable safety limits for rehabilitation devices pending human-cell validation. Third, actin orientation metrics merit exploration as biomarkers for mechanical overloading in myopathies.

Several limitations warrant acknowledgment. First, the single-cell FE model neglects intermediate filaments and microtubules [6,29] which are also critical components for nuclear mechanotransduction. This omission potentially compromises nuclear strain accuracy despite membrane strain validation [16], as demonstrated by Khounsaraki et al. [31]. Second, the 60 min experimental window precludes observation of long-term adaptations (e.g., 7-day chondrogenesis [11]). Third, the universal 20% membrane strain threshold requires cell-type-specific calibration; epithelial cells, for instance, tolerate strains up to 110% [32]. Additionally, device constraints prevent multicellular or multiaxial loading. In particular, while our hydrogel-based loading interface is expected to reduce hydrogel–cell adhesion artifacts, we did not directly quantify adhesion-related phenomena in this study. Future work is needed to rigorously compare adhesion responses between this device and conventional rigid-substrate systems. Most critically, molecular actuators governing actin reorientation (e.g., Piezo1) remain uncharacterized, impeding mechanistic understanding.

## 5. Conclusions

In this study, we developed a programmable cyclic compression device and combined in vitro experiments with a 3D finite element model to investigate how uniaxial compression affects C2C12 myoblasts. Static compression induced actin filament reorientation toward a more perpendicular alignment with the cell long axis, which increased maximum principal strain on the cell membrane and cell mortality. Conversely, cyclic compression reversed this actin reorientation, reduced membrane tensile strain, and improved cell viability, with optimal cytoprotection at 0.25 Hz. These findings suggest that actin filament orientation is an important mediator linking mechanical loading to membrane strain distribution, which may inform the design of mechanical stimulation strategies in tissue engineering and regenerative medicine applications.

## Figures and Tables

**Table 1:** Material properties of the nucleus and cytoplasm complex.

Component	G_0 (kPa)	G_∞ (kPa)	Poisson’s Ratio	Element Number	Relaxation Time Constant (s)
Nucleus	3.8	2.0	0.3	14,551	0.3
Cytoplasm complex	1.8	0.9	0.37	7393	0.3

## Data Availability

The data generated during this study are available from the corresponding author upon reasonable request.

## Supplementary Materials

The following supporting information can be downloaded at: https://www.mdpi.com/article/10.3390/bioengineering12121317/s1, Figure S1: Representative fluorescence images and images with detected actin filaments under 0 min, 10 min, 20 min, 30 min and 60 min static compression. Figure S2: Representative fluorescence images and images with detected actin filaments under 0 min, 20 min and 60 min 0.25 Hz cyclic compression. Figure S3: Representative fluorescence images and images with detected actin filaments under 0 min, 20 min and 60 min 0.5 Hz cyclic compression. Figure S4: Representative fluorescence images and images with detected actin filaments under 0 min, 20 min and 60 min 1 Hz cyclic compression. Figure S5: Representative fluorescence images and images with detected actin filaments under 0 min, 20 min and 60 min 10 Hz cyclic compression. Figure S6: Contour plots of the maximum principal strain distribution and its peak value on cell membrane under 20 min static and cyclic compression. Figure S7: Contour plots of the maximum principal strain distribution and its peak value on cell membrane under 60 min static and cyclic compression.

## Abbreviations

The following abbreviations are used in this manuscript:

FE	Finite element
BM-MSCs	Bone marrow mesenchymal stem cells
