# Supplementary material for: In Vitro Experimental and Numerical Simulation Study on the Influence of Uniaxial Cyclic Compression on Cytoskeletal Structure
Source: Bioengineering (Basel). 2025 Dec 2;12(12):1317. doi: 10.3390/bioengineering12121317 (PMC12729777; doi:10.3390/bioengineering12121317)
Supplement: Supplementary file 1 [file bioengineering-12-01317-s001.zip › bioengineering-3986926-supplementary.pdf]

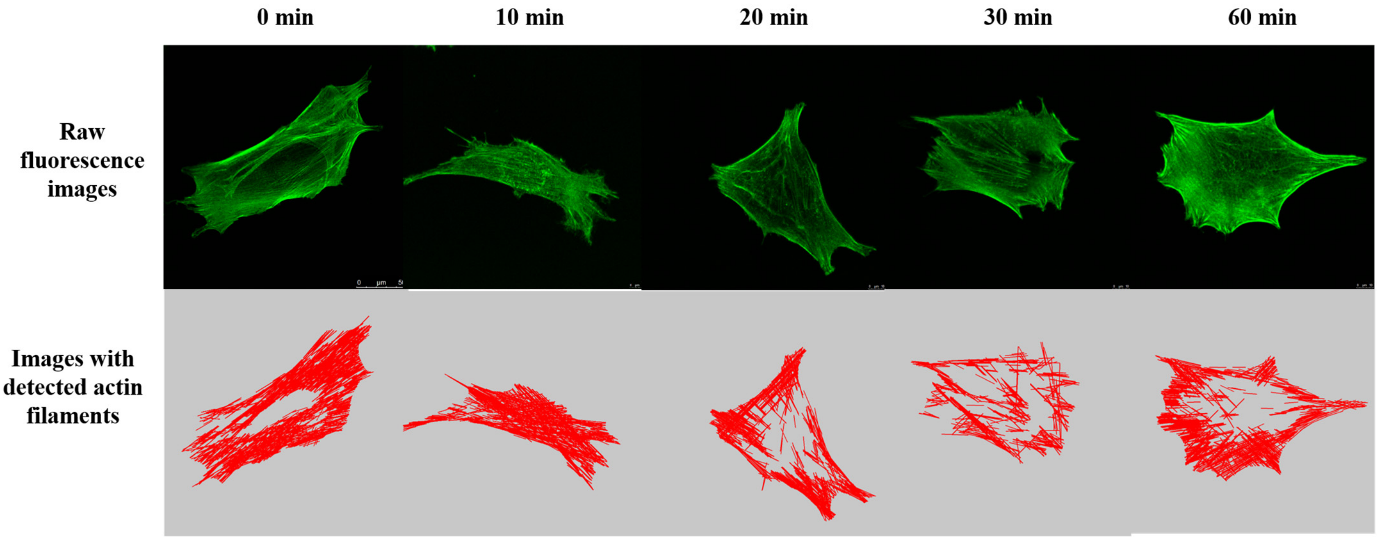

Figure S1. Representative fluorescence images and images with detected actin filaments under 0 min, 10 min, 20 min, 30 min and 60 min static compression.

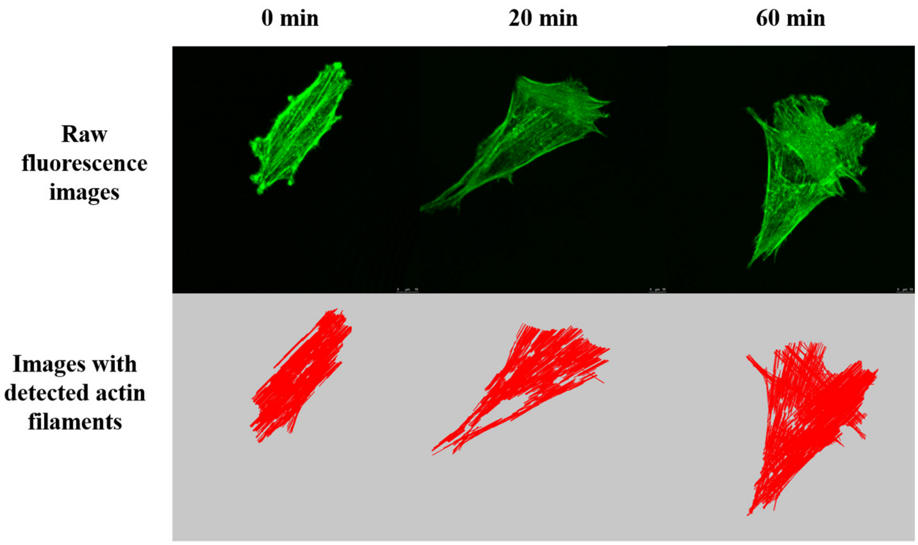

Figure S2. Representative fluorescence images and images with detected actin filaments under 0 min, 20 min and 60 min 0.25Hz cyclic compression.

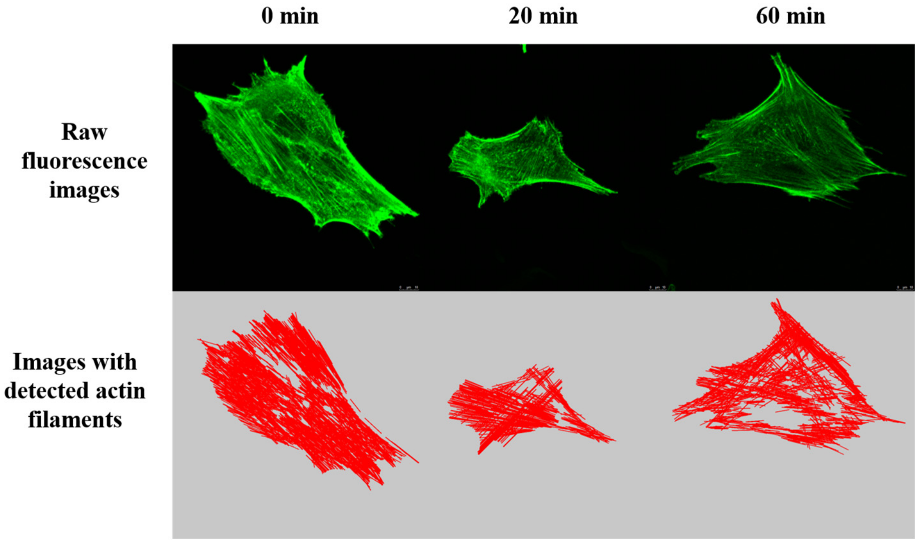

Figure S3. Representative fluorescence images and images with detected actin filaments under 0 min, 20 min and 60 min 0.5Hz cyclic compression.

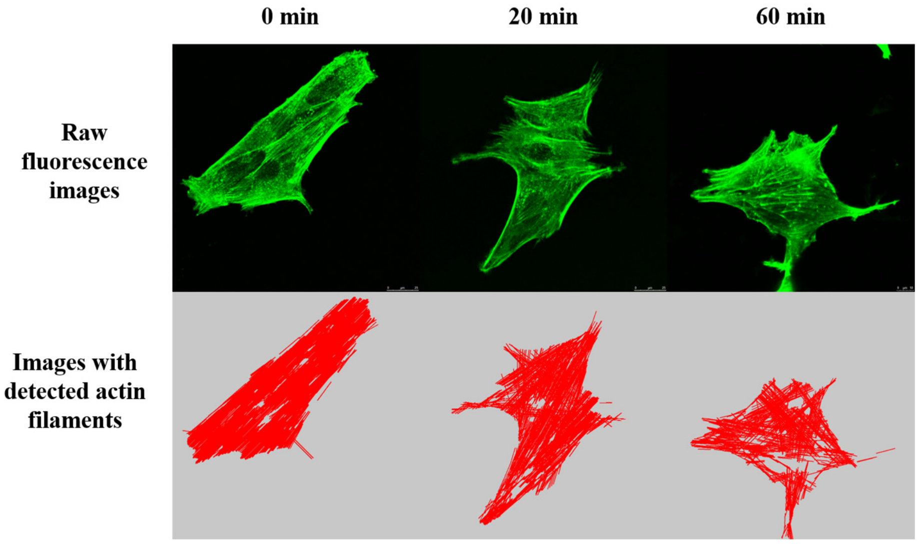

Figure S4. Representative fluorescence images and images with detected actin filaments under 0 min, 20 min and 60 min 1Hz cyclic compression.

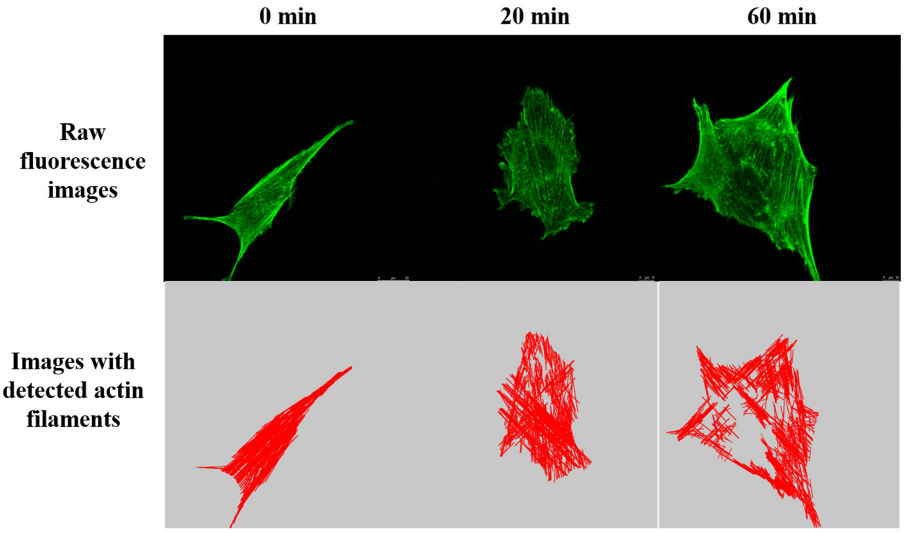

Figure S5. Representative fluorescence images and images with detected actin filaments under 0 min, 20 min and 60 min 10Hz cyclic compression.

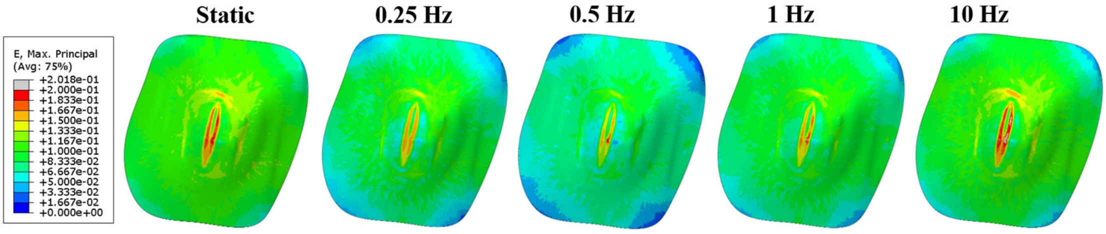

Figure S6. Contour plots of the maximum principal strain distribution and its peak value on cell membrane under 20 min static and cyclic compression.

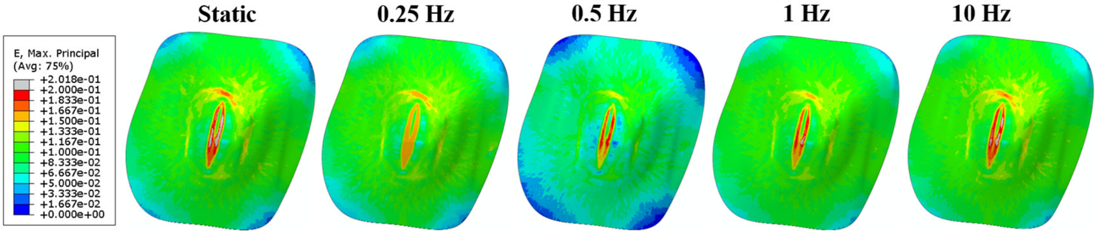

Figure S7. Contour plots of the maximum principal strain distribution and its peak value on cell membrane under 60 min static and cyclic compression.
